# Concordance of microbial and visual health indicators of white-band disease in nursery reared Caribbean coral *Acropora cervicornis*

**DOI:** 10.7717/peerj.15170

**Published:** 2023-06-21

**Authors:** Monica D. Schul, Dagny-Elise Anastasious, Lindsay J. Spiers, Julie L. Meyer, Thomas K. Frazer, Anya L. Brown

**Affiliations:** 1 Department of Soil, Water, and Ecosystem Sciences, University of Florida, Gainesville, FL, United States of America; 2 Little Cayman Research Center, Central Caribbean Marine Institute, Little Cayman, Cayman Islands; 3 School of Fisheries, University of Florida, Gainesville, FL, United States of America; 4 Fish & Wildlife Research Institute, Florida Fish & Wildlife Conservation Commission, Marathon, FL, United States of America; 5 College of Marine Science, University of South Florida, St. Petersburg, FL, United States of America; 6 School of Natural Resources and Environment, University of Florida, Gainesville, FL, United States of America; 7 Bodega Marine Lab, Department of Evolution and Ecology, University of California, Davis, Bodega Bay, CA, United States of America

**Keywords:** *Acropora cervicornis*, Core microbes, Pathobiome, Coral nursery, Coral disease, White band disease, Coral reefs

## Abstract

**Background:**

Coral diseases are one of the leading causes of declines in coral populations. In the Caribbean, white band disease (WBD) has led to a substantial loss of *Acropora* corals. Although the etiologies of this disease have not been well described, characterizing the coral microbiome during the transition from a healthy to diseased state is critical for understanding disease progression. Coral nurseries provide unique opportunities to further understand the microbial changes associated with diseased and healthy corals, because corals are monitored over time. We characterized the microbiomes before and during an outbreak of WBD in *Acropora cervicornis* reared in an ocean nursery in Little Cayman, CI. We asked (1) do healthy corals show the same microbiome over time (before and during a disease outbreak) and (2) are there disease signatures on both lesioned and apparently healthy tissues on diseased coral colonies?

**Methods:**

Microbial mucus-tissue slurries were collected from healthy coral colonies in 2017 (before the disease) and 2019 (during the disease onset). Diseased colonies were sampled at two separate locations on an individual coral colony: at the interface of Disease and ∼10 cm away on Apparently Healthy coral tissue. We sequenced the V4 region of the 16S rRNA gene to characterize bacterial and archaeal community composition in nursery-reared *A. cervicornis*. We assessed alpha diversity, beta diversity, and compositional differences to determine differences in microbial assemblages across health states (2019) and healthy corals between years (2017 and 2019).

**Results:**

Microbial communities from healthy *A. cervicornis* from 2017 (before disease) and 2019 (after disease) did not differ significantly. Additionally, microbial communities from Apparently Healthy samples on an otherwise diseased coral colony were more similar to Healthy colonies than to the diseased portion on the same colony for both alpha diversity and community composition. Microbial communities from Diseased tissues had significantly higher alpha diversity than both Healthy and Apparently Healthy tissues but showed no significant difference in beta-diversity dispersion. Our results show that at the population scale, Healthy and Apparently Healthy coral tissues are distinct from microbial communities associated with Diseased tissues. Furthermore, our results suggest stability in Little Cayman nursery coral microbiomes over time. We show healthy Caymanian nursery corals had a stable microbiome over a two-year period, an important benchmark for evaluating coral health via their microbiome.

## Introduction

Globally, coral reefs are disappearing at an alarming rate due to multiple stressors that are leading to declines in coral populations (Pandolfi et al., 2003; Diaz et al., 2016; Pollock et al., 2019). Coral diseases are an increasingly significant contributor to reef decline, particularly in the Caribbean (Weil, 2004; Harvell et al., 2007). Specifically, white band disease (WBD) has contributed to the death of over 80% of critical habitat-building corals, *Acropora palmata* and *Acropora cervicornis* (Aronson & Precht, 2001). These losses have led to the listing of both *Acropora* species as threatened under the U.S. Endangered Species Act (Guertin, 2014) and critically endangered under the International Union for Conservation of Nature (IUCN) Red List (Aronson et al., 2008).

 WBD was initially identified in the late 1970s and was originally described as a line of tissue necrosis and tissue sloughing that progresses from the middle of the colony and advances out toward the branches, leaving behind bare skeleton which is quickly colonized by turf algae (Gladfelter et al., 1977; Gladfelter, 1982). Although suspected to be bacterial even then, now we rely on information from current outbreaks of disease throughout the Caribbean to understand the etiology of the disease (Gil-Agudelo, Smith & Weil, 2006; Williams & Miller, 2005). However, describing current outbreaks can be difficult because at least four acute tissue loss diseases have been described, including WBD Type I & II, rapid tissue loss, and shut down reaction, which all show similar signs of disease, and have been previously suggested to be collectively referred to as WBD (Gignoux-Wolfsohn et al., 2020).

Although WBD has been present since the 1970s, identifying potential pathogens has been tenuous. Over time several candidate pathogens have been suggested, including *Vibrio charchariae*/*harveyi* (WBD Type II, Ritchie & Smith, 1998); *Bacillus* sp., *Lactobacillus suebicus* (Sweet, Croquer & Bythell, 2014) and bacteria in the order Rickettsiales (Gignoux-Wolfsohn & Vollmer, 2015). However, recent studies, particularly since the advent and ubiquity of next-generation sequencing methods, suggest that, like other coral diseases, WBD may not follow Koch’s and Hill’s fundamental postulates that indicate that a single pathogen is responsible for the disease (Sweet & Bulling, 2017); (Vega Thurber et al., 2020). Instead, like many coral diseases, WBD may be polymicrobial (Sweet, Croquer & Bythell, 2014) or a co-infection with ciliates (Verde, Bastidas & Croquer, 2016), suggesting that instead the disease is perhaps best characterized by a pathobiome—a disease-causing microbial phenotype (Sweet & Bulling, 2017). Transmission studies and surveys suggest the WBD consortia may include bacterial taxa from the orders Rickettsiales, Vibrionales, Alteromonadales, and Flavobacteriales, which vary with site and environmental conditions (Gignoux-Wolfsohn & Vollmer, 2015; Certner et al., 2017; Rosales et al., 2019).

In WBD and other coral diseases, microbial signatures of disease can be evident next to the lesion (Pantos & Bythell, 2006; Meyer et al., 2016; Meyer et al., 2019; Gignoux-Wolfsohn, Aronson & Vollmer, 2017). However, they can also be present on non-diseased parts of the tissue as a transitional community, distinct from healthy and diseased tissues (*e.g.*, in Pacific and Caribbean acroporid diseases; Pollock et al., 2016; Pantos & Bythell, 2006). Additionally, even healthy colonies may harbor disease agents or early warning signs during an outbreak, particularly if the pathogen (or pathogens) are waterborne (Gignoux-Wolfsohn, Marks & Vollmer, 2012). The newly described stony coral tissue loss disease outbreak also shows this pattern (Rosales et al., 2020). However, sampling before disease affects a population is rare, as there are few studies that monitor coral microbiomes, and there are often no visual indicators that disease is imminent. Thus, characterizing the disease and the changes corals experience in their microbiome over time and across a colony is critical in understanding WBD and other acute tissue loss diseases.

Monitoring coral colony health and the microbiomes associated with these colonies are increasingly more feasible due to coral nurseries. To restore populations of acroporid corals, many organizations throughout the Caribbean are using a coral-gardening approach to propagate corals through colony fragmentation, followed by growth in ocean-based nurseries, before transplanting back onto degraded reefs (Johnson, Lustic & Bartel, 2011). Like wild corals, nursery corals are exposed to stressors such as heat, eutrophication, inclement weather, and disease (Johnson, Lustic & Bartel, 2011; Young, Schopmeyer & Lirman, 2012; Rosales et al., 2019). Nurseries provide ideal conditions in which disease can be studied, with historical information on the reared corals that can help identify epidemiological clues that were missed during earlier disease outbreaks in wild populations. 

We characterized the early microbial signatures underlying a WBD outbreak in a population of *A. cervicornis* reared in a coral nursery in Little Cayman, Cayman Islands. During this outbreak, the disease presented as a bright white band of denuded skeleton advancing towards the coral tips, where tissue was actively sloughing off and the white skeleton was recolonized by algal turfs. To understand how disease influenced microbial communities within and among colonies, we characterized differences among bacterial communities associated with healthy coral colonies (no visual signs of disease), lesions on diseased corals, and apparently healthy tissue on diseased coral colonies at the beginning of the outbreak. Because coral microbial communities often differ from healthy to disease (Sunagawa et al., 2009; Ushijima et al., 2012; Arotsker et al., 2015; Rosales et al., 2019), we expected patterns of composition and diversity to differ between Healthy and Diseased tissues. We expected that Apparently Healthy samples on Diseased colonies would show either (1) no differences in bacterial diversity and community composition compared to healthy samples, (2) no differences in diversity and community composition compared to Disease samples, or (3) an intermediate community as it transitions from a healthy to a symptomatic diseased coral segment. Additionally, we compared microbial community composition and diversity of healthy coral colonies in 2019 to samples from 2017, when all colonies were healthy in the nursery population, to capture the variation or stability in healthy *Acropora cervicornis* microbiomes before *versus* during a disease outbreak.

## Materials & Methods

### Nursery

Samples were collected from corals in the Central Caribbean Marine Institute’s (CCMI) coral nursery in Little Cayman, CI at 18m depth. Sample collections were completed under permits approved by the Cayman Islands Department of Environment for Anya Brown (mucus sampling) in 2019, and for Carrie Manfrino and Thomas Frazer in 2018–2020 (the ongoing coral nursery permit). A CITES export permit was approved in May 2019 with approval number 2019/KY/001011. The coral nursery contained five different coral genotypes, differentiated in the nursery by colored beads (black, blue, green, red, and yellow). Genotype designations were based on the original donor colonies that were collected in 2016 (Drury et al., 2017; Maneval et al., 2021). Fragments were suspended from 1.5 m × 3 m PVC frames approximately 1 m above the benthos (Maneval et al., 2021). Frames were located ∼1 m apart within a sandy groove. Each frame contained coral colonies (∼30) from the same genotype.

### Sample collection—2019

**Figure 1:**
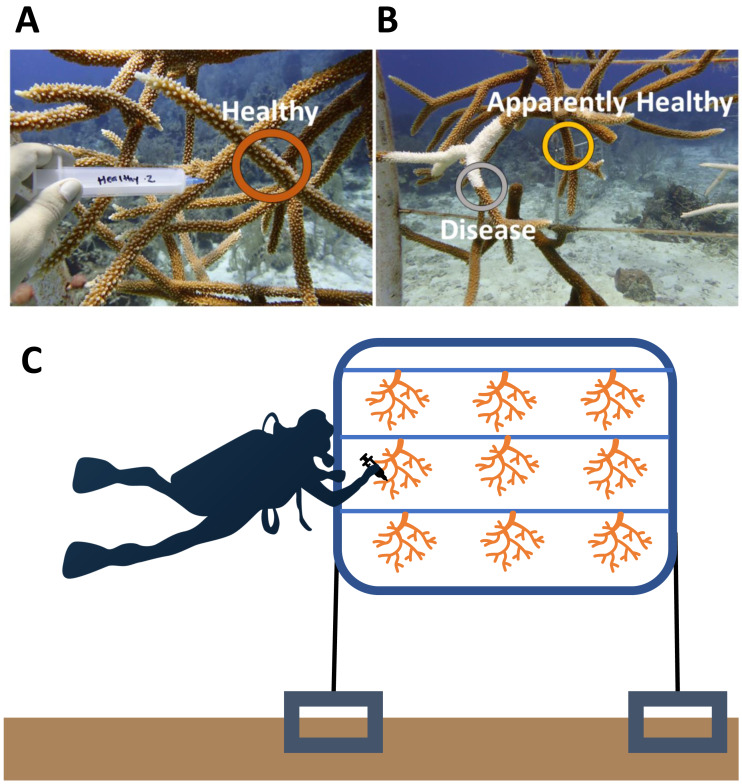
Representative sampling of *Acropora cervicornis* colonies in Little Cayman, CI. (A) Healthy (dark orange circle) coral mucus/tissue slurry samples were taken haphazardly on a coral colony that showed no visual signs of disease or tissue loss. (B) Disease (gray circle) and Apparently Healthy (light orange circle) sampling sites for coral mucus/tissue slurry from the nursery reared *A. cervicornis* in 2019. Disease shows a distinct white band of exposed coral skeleton adjacent to live coral tissue. Apparently Healthy samples were chosen on the same coral colony 10 cm away from a disease lesion. (C) A graphic representation of sampling of colonies in a group (samples representing all the treatments from one frame). Each frame consisted of colonies from one genotype. In this study, colonies sampled from the same frame were in the same genotype in all 2019 samples.

The disease signs began in early May 2019 and peaked in July 2019 (Brown et al., 2022). We sampled the microbial communities of the corals shortly after the onset, when only a subset of corals in the entire nursery showed signs of disease. Samples from 10 healthy and 10 diseased corals were collected on 16 May 2019 from across the nursery population. We sampled the surface of corals (mucus and some tissue) using sterile 20 ml needleless syringes. For each tissue sample, we first agitated the coral surface lightly with the tip of the syringe and then collected the coral mucus produced. On a healthy coral, the sample area was chosen haphazardly (Fig. 1A), as microbial communities are similar across whole healthy *A. cervicornis* colonies (Miller et al., 2020). On diseased coral colonies, we sampled two different tissue types using separate syringes: one at the disease front adjacent to exposed skeletal tissue (Disease) and the other ∼10 cm away from the disease front on Apparently Healthy tissue (Fig. 1B). Corals were sampled in groups (*i.e.,* statistical blocks) to account for environmental factors that may also lead in the coral microbial communities. Each group (*n* = 10 groups) included the two tissue samples from the diseased coral colony, and a visibly healthy colony of the same genotype (and on the same frame, *e.g.*, Fig. 1C). A complete schematic showing the layout of the nursery, including the frames sampled here is available in Brown et al. (2022). Colonies in 2019 were sampled across five genotypes to provide a general description of the microbial changes associated with disease for nursery coral population (2 black, 3 blue, 1 green, 2 red, and 2 yellow). We did not explicitly sample to test for genotypic differences, instead, we focused on the disease signature of the corals in the nursery population for colonies that were diseased as the outbreak began.

### Sample collection—2017

Two years prior to the disease, microbial samples were taken from some of the colonies (and replicated in genotypes) within the nursery (Miller et al., 2020). Because these samples were collected before the outbreak, we had the unique opportunity to compare microbial communities in this population before and after a disease outbreak. In brief, in December 2017 nine visibly healthy corals across three genotypes (3 green, 3 red, and 3 yellow) were collected and processed in the same manner as in the current study, with the exception that all nine colonies were grown on the same frame (Fig. 1C) (Miller et al., 2020). A total of nine samples, one from each colony, were randomly selected from these healthy coral colonies (Healthy 2017) to compare to Healthy colonies in 2019 (*n* = 9).

### Sample processing

All syringes with coral mucus samples were placed on ice after collection and transported back to CCMI. Mucus was allowed to settle to the bottom of each syringe and then expelled into a 2 ml cryogenic vial and spun down to a pellet (∼0.2 µl) with an Eppendorf Minispin centrifuge (5 mins, at max speed). After decanting the excess seawater, we added 1 ml of RNAlater (Ambion, Austin, TX), left samples on the bench overnight, and then froze at −20 °C. Frozen samples were transferred to the University of Florida for sample processing and analysis.

### Laboratory preparation

Prior to DNA extraction, we centrifuged samples at 10,000 *g* for 5 min and removed the RNAlater using a micropipette. We extracted the mucus DNA with the DNeasy PowerSoil Kit (Qiagen, Germantown, MD) following the manufacturer’s protocol. Extracted DNA was then checked for DNA concentrations on a Denovix DS-11 FX + fluorometer (Denovix,Wilmington, DE) preceding PCR amplification.

We amplified the V4 region of the 16S rRNA gene using the 515F primer (Parada, Needham & Fuhraman, 2016) and 806RB primer (Apprill et al., 2015) according to the Earth microbiome protocol (Gilbert, Jansson & Knight, 2014). Each 26.75 µl PCR reaction contained 12.5 µl of Phusion High-fidelity Master Mix (New England Biolabs, Ipswich, MA), 1.25 µl of 5 µM of each primer, 0.75 µl of dimethyl sulfoxide (DMSO), 9 µl of PCR grade water, and 2 µl of DNA template. PCR amplification was performed under the following conditions: 94 °C for 3 min, 35 cycles of 94 °C for 45 s, 50 °C for 1 min, 72 °C for 90 s, and a final elongation step at 72 °C for 10 min. Triplicate PCR products were consolidated and cleaned with the MinElute PCR purification kit (Qiagen), and the concentration of purified PCR products was quantified with a Denovix. A total of 240 ng of each library was submitted to the University of Florida Interdisciplinary Center for Biotechnology Research (RRID:SCR_019152) for sequencing on an Illumina MiSeq with paired 150-bp reads.

### Bioinformatics and statistics

Primers and adapters were removed from raw sequencing reads with cutadapt *v.* 1.8.1 (Martin, 2011). We used the DADA2 pipeline (version 1.16.0, Callahan et al., 2016) in R 3.6.3 (R Core Team) for quality control, merging sequences, and assigning ASVs (amplicon sequence variants). Reads were quality filtered separately for each run using the following parameters: filterAndTrim (fnFs, filtFs, fnRs, truncLen = c(150,150), maxN = 0, maxEE = (c(2,2), truncQ = 2, rm.phix = TRUE, compress = TRUE, multithread = TRUE)). The sequences from the 2019 samples (*n* = 30) and the select 2017 samples (*n* = 9) from Miller et al. (2020) were merged to obtain the full denoised ASVs. The 2019 samples are available in the NCBI SRA database under the BioProject PRJNA679809 and the 2017 samples are available in PRJNA308473 (Table S1). ASVs provide an exact sequence and allow for one base pair variation (Callahan et al., 2016). Chimeras were removed and taxonomy was assigned to the sequence variants using SILVA version 132 small subunit ribosomal RNA database (Yilmaz et al., 2014). ASVs, taxa, and metadata tables were imported into phyloseq (McMurdie & Holmes, 2013). Sequences classified as eukaryotes, chloroplasts, or mitochondria were removed before further analysis of bacterial and archaeal communities.

To determine differences in microbial diversity, we performed a rarefaction curve (with a step size of 100) on our dataset (Fig. S1), and then estimated the Shannon diversity index and the Inverse Simpson index on unrarefied data as the rarefaction curves by sample saturated in phyloseq (Fig. S1). Shannon diversity is more sensitive to rare taxa, whereas the Inverse Simpson Index is influenced by dominant taxa (Alberdi & Gilbert, 2019).

To understand differences in beta diversity (*i.e.,* variability across samples), we calculated the Bray Curtis dissimilarity among our samples using the relative abundances of ASVs. We then estimated beta-diversity dispersion using this matrix by estimating the distance to a group’s (*i.e.,* level within a treatment) centroid from each sample. This measure of multivariate dispersion was calculated using the betadisper function in vegan (Oksanen et al., 2019) based on tissue type (Healthy, Disease, and Apparently Healthy) for the 2019 samples and year (2017 *vs* 2019) for the Healthy colonies.

For each alpha diversity and beta diversity measure, we used separate linear models to compare tissue type in 2019 (Healthy, Diseased, Apparently Healthy) and our blocking factor (group). Model residuals were plotted to visually test for normality. When models were significant (*p* < 0.05) for treatment, we performed Tukey HSD post hoc tests to determine significant differences among levels in the treatment. For healthy samples from 2017 and 2019, we also used linear models, comparing year with genotype (as a blocking factor) as a fixed effect to consider the same genotype across years.

We examined differences in community composition first by visualizing the data with a principal coordinates analysis (PCoA) using ggplot2 and ggforce packages (Wickham, 2016; Pedersen, 2022) based on the Bray-Curtis distance matrix described above. To determine if microbial communities associated with tissue types differed significantly, we performed a permutational analysis of variance (PERMANOVA) using the adonis function in vegan (Oksanen et al., 2019). We compared the coral health status (tissue type) at 999 permutations, and treated group as a blocking term (*i.e.,* strata). We separately tested for differences in healthy corals across years with a PERMANOVA on the healthy-only Bray-Curtis dissimilarity matrix, comparing across years and including genotype as a blocking term.

To understand which taxa led to differences between treatments, we used two differential abundance approaches, an Analysis of Communities (ANCOM) based on bacterial ASVs (Mandal et al., 2015) and DESeq2 (Love, Huber & Anders, 2014). We used ANCOM to compare treatments with more than two levels (2019 health statuses). ANCOM uses the Aitchison’s log ratio to iteratively compare taxa. W statistics greater than or equal to 90% of the total number of genera tested were considered at an alpha level of 0.05. Because DESeq2 can be more robust to small samples (Weiss et al., 2017), we ran a DESeq2 analysis comparing Healthy and Disease samples (from 2019), and healthy samples across years (2017 *vs* 2019). With DESeq2, we used raw sequence counts to estimate size factors, or normalization factors for our samples. Wald tests were used to calculate which ASVs were significantly differentially abundant using a Negative Binomial generalized linear model on the effect size (log2fold ratio) and dispersion outputs. To compare the healthy samples between years, we removed the dominant ASVs (in the order Rickettsiales), to compare the non-dominant ASVs associated with healthy coral colonies.

## Results

**Figure 2:**
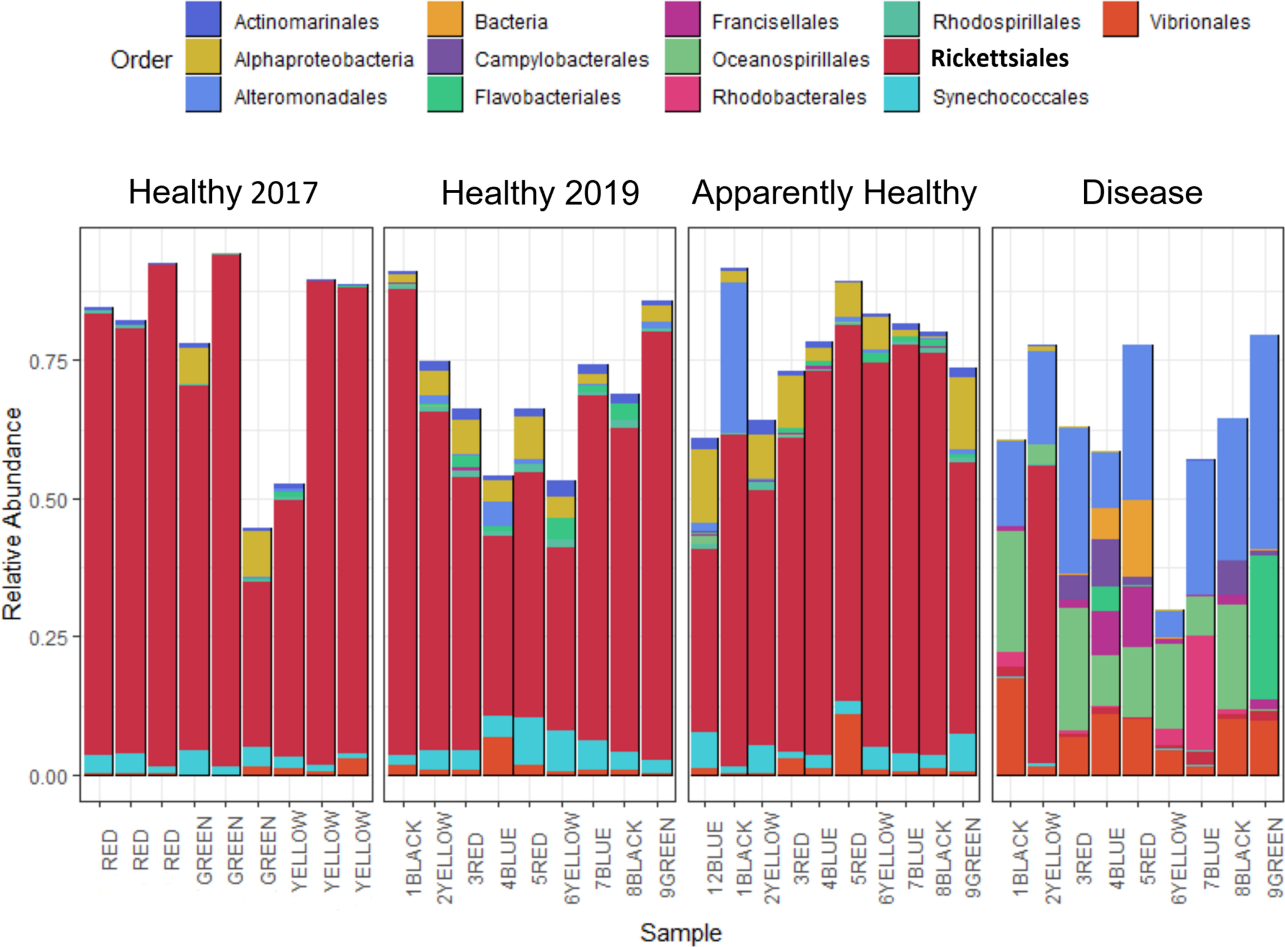
Relative abundance of bacterial and archaeal taxa for *A. cervicornis* mucus samples taken in 2017 and 2019 represented at the order level. Each bar represents a different coral. Only samples with greater than 800 sequences are included in this plot, and ASVs with relative abundances greater than 0.5%. Samples from 2019 included “Healthy”, “Apparently Healthy”, and “Disease” tissue types. Colors indicate Order designated by the SILVA database. Both Healthy 2017 and 2019 samples and 2019 Apparently Healthy samples appear similar in bacterial composition, mainly due to the high relative abundance of the bacterial order Rickettsiales (in bold). Disease samples appear distinct from all other samples in composition and abundance of taxa. The *x*-axis of the graph shows each sample’s blocking group and genotype designation (indicated by colors red, green, yellow, black, and blue). Samples from 2017 were collected from different colonies (and groups) than in 2019.

After quality filtering and removal of chloroplasts, eukaryotes, and mitochondrial sequences, samples had 1,127 to 232,727 sequences per sample with an average of 80,392 per sample (including the 2017 samples, Table S1). Two samples with less than 800 sequencing reads were not used for data analysis, including one Healthy 2019 tissue type and one Disease tissue type sample. After the filtering steps, we had 37 total samples: (Healthy 2017 *n* = 9, Healthy 2019 *n* = 9, Apparently Healthy *n* = 10, and Disease *n* = 9). Healthy colonies and Apparently Healthy tissues had high relative abundances of ASVs in the order Rickettsiales (average abundance ± se in healthy colonies = 56% ± 6% in 2019 and 75% ± 6% in 2017; and Apparently Healthy tissues = 60% ± 4%). Comparatively, Disease tissues showed lower abundances of Rickettisales (average abundance = 7% ± 6%; Fig. 2). One genus was most abundant within this order: MD3-55 (75% in Healthy tissues in 2017, 56% in Healthy tissues in 2019, 60% in Apparently Healthy tissues, and 7% in Disease tissues).

### Alpha diversity

**Figure 3:**
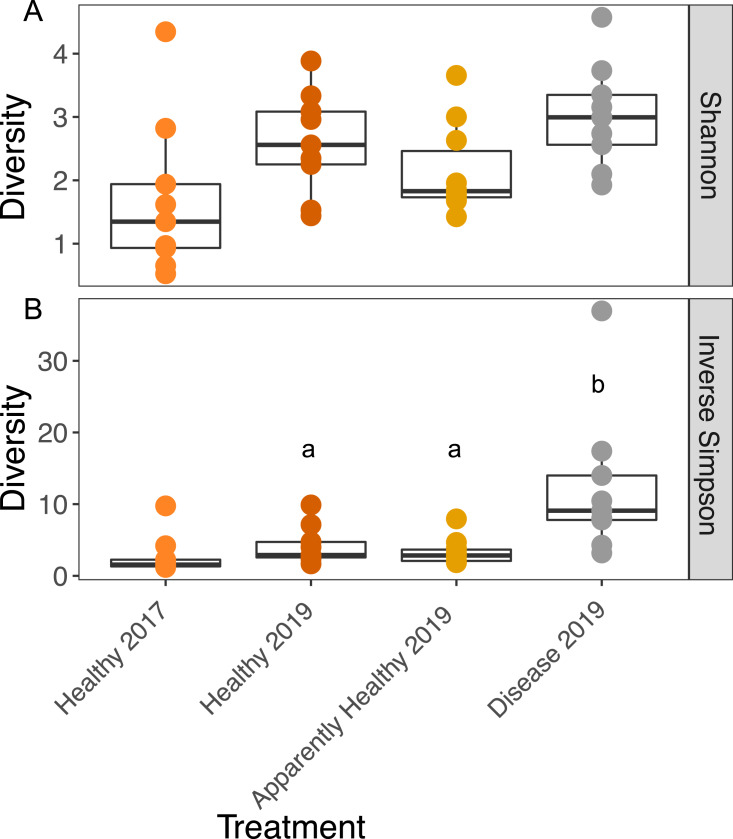
Boxplots of the Shannon and Inverse Simpson diversity of microbial communities in *A. cervicornis* based on tissue type and year. Each point represents a single tissue sample. Colors represent tissue types and year. The center bar of each box plot represents median diversity. Whiskers extend to ±1.5 of the inter-quartile range. (A) Shannon diversity of microbial communities was not significantly different across tissue types or years in *A. cervicornis*. (B) The Inverse Simpson index was not significant across Healthy tissue types for each year, but within differed significantly across tissues treatment types in 2019. Disease samples show higher Inverse Simpson diversity than either Healthy or Apparently Healthy tissue types in 2019. Significant differences (*p* < 0.05) based on Tukey HSD tests are indicated by different letters. Healthy samples from 2017 and 2019 were not significantly different for either alpha diversity measurement.

We observed no significant differences in Shannon diversity among 2019 tissue types (Tissue type: *F*_2,16_ = 3.26, *p*-value = 0.06), when we included group as a blocking factor (to account for frame and coral genotype; group: *F*_9,16_ = 1.3, *p* = 0.30), or when we dropped group from the analysis (Tissue type: *F*_2,25_ = 2.933, *p*-value = 0.07, Fig. 3A).

We calculated the Inverse Simpson Index to test differences in dominance across the tissue types. Diseased corals showed significantly higher Inverse Simpson Indices compared to both healthy (Inverse Simpson: Tissues Type: *F*_2,16_ = 7.63, *p*-value = 0.004; Tukey HSD: *p* = 0.016); and Apparently Healthy corals (Tukey HSD: *p* = 0.006), indicating these tissues samples were not dominated by a single group (Fig. 3). However, Healthy and Apparently Healthy tissues were not different from each other (Tukey HSD: *p* = 0.93). Group (the blocking effect) did not differ significantly (Inverse Simpson: Group: *F*_9,16_ = 1.65, *p*-value = 0.18).

Among Healthy tissues in 2017 and 2019, Shannon diversity did not differ significantly across years (Shannon diversity: Year: *F*_1,12_ = 2.79, *p*-value = 0.12, Fig. 3A) or from the blocking factor (Genotype: *F*_4,12_ = 0.2, *p*-value = 0.94). We also did not find differences in the Inverse Simpson index among Healthy tissue types across years (Inverse Simpson Year: *F*_1,12_ = 1.08, *p*-value = 0.32, Fig. 3B, Genotype: *F*_4,12_ = 0.33, *p*-value = 0.82).

### Beta-diversity dispersion

**Figure 4:**
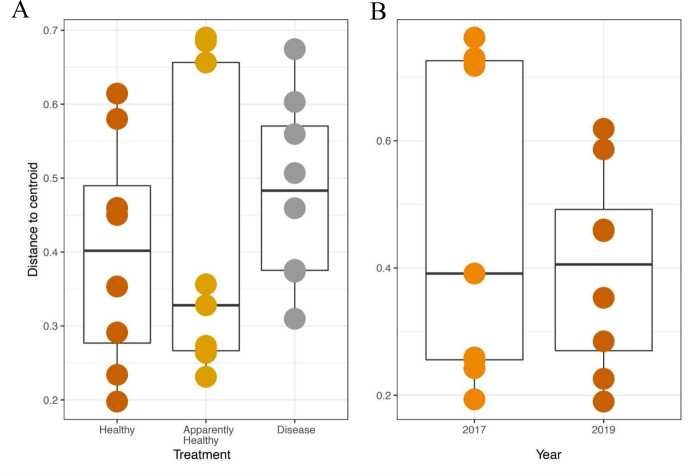
Beta diversity dispersion in *A. cervicornis* samples for 2019 tissue types and Healthy tissues across years. Beta diversity (dispersion) was measured and plotted for (A) Healthy, Apparently Healthy, and Disease samples in 2019 and (B) Healthy corals across years. Each point represents the distance to a centroid value for each sample within a treatment. Middle bars on each plot represent the median distance to centroid for each sample type. Upper and lower ends of each box plot represent the upper and lower range of the data set, respectively. Microbial variance was not significantly different across (A) tissue treatment types for 2019 or (B) Healthy tissues across years.

**Figure 5:**
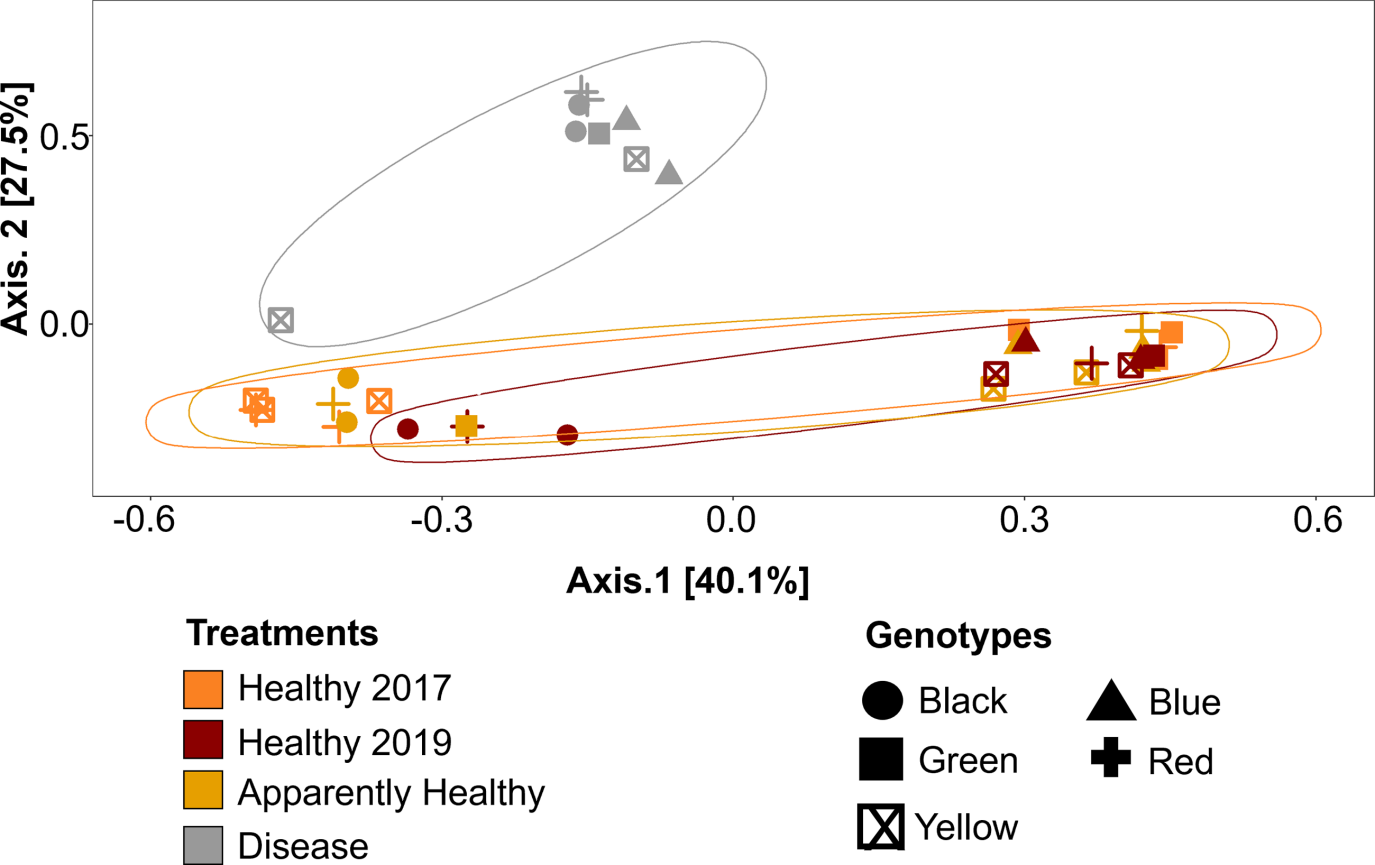
Principal Coordinate Analysis (PCoA) of *A. cervicornis* microbial communities for 2017 and 2019 tissue types. Principle coordinate analysis based on the Bray Curtis dissimilarity on the relative abundances of ASVs in each sample. Each point represents an individual coral mucus sample. The color of each ellipse represents tissue types and year, and the shape of the point represents genotype. Ellipses were created using the geom_mark_ellipse() function in ggplot. Points that are closer together indicate communities that are more similar. Disease tissues typically clustered together (top left ellipse), indicating disease samples were more similar to one another but different from Healthy or Apparently Healthy corals. Healthy and Apparently Healthy samples clustered near each other. PERMANOVA results suggest disease tissues are different from Healthy tissues in 2019. Between years, PERMANOVA results suggest no differences in Healthy tissues (*p*-value =0.119) and no difference in genotypes (*p*-value =0.11).

We expected Disease tissues to be more variable than Healthy tissues, however, they did not show statistically higher beta-diversity dispersion than Healthy tissues (Tissue Type: *F*_2,16_ = 1.45, *p*-value = 0.26, Group: *F*_9,16_ = 0.42, *p*-value = 0.91, Figs. 4A and 5). We also did not observe any significant differences in dispersion across years for Healthy samples (*F*_1,12_ = 1.25, *p* = 0.28, Fig. 4B), nor any significant differences in dispersion among genotypes of Healthy corals (*F*_4,12_ = 2.8, *p* = 0.07).

### Compositional differences

Microbial communities differed depending on tissue type (PERMANOVA: *p*-value = 0.001, R^2^ = 0.35, Fig. 5). Disease tissue samples were more similar to one another than to Healthy or Apparently Healthy tissue samples (Fig. 5). Healthy 2019, Healthy 2017, and Apparently Healthy tissue samples showed little variation from one another (Fig. 5). We did observe differentiation across PCoA axis 1 (40% of the explained variation), which was associated with the healthy coral microbiome. We expect this may be due to an interaction with health status (Healthy and Apparently Healthy) and genotype that we are unable to resolve with this dataset (Fig. S2), as some Healthy and Apparently Healthy samples from the same genotype tended to cluster on either side of the PCoA’s first axis (*e.g.*, the Healthy and Apparently Healthy communities in the red genotype are in both clusters).

**Figure 6:**
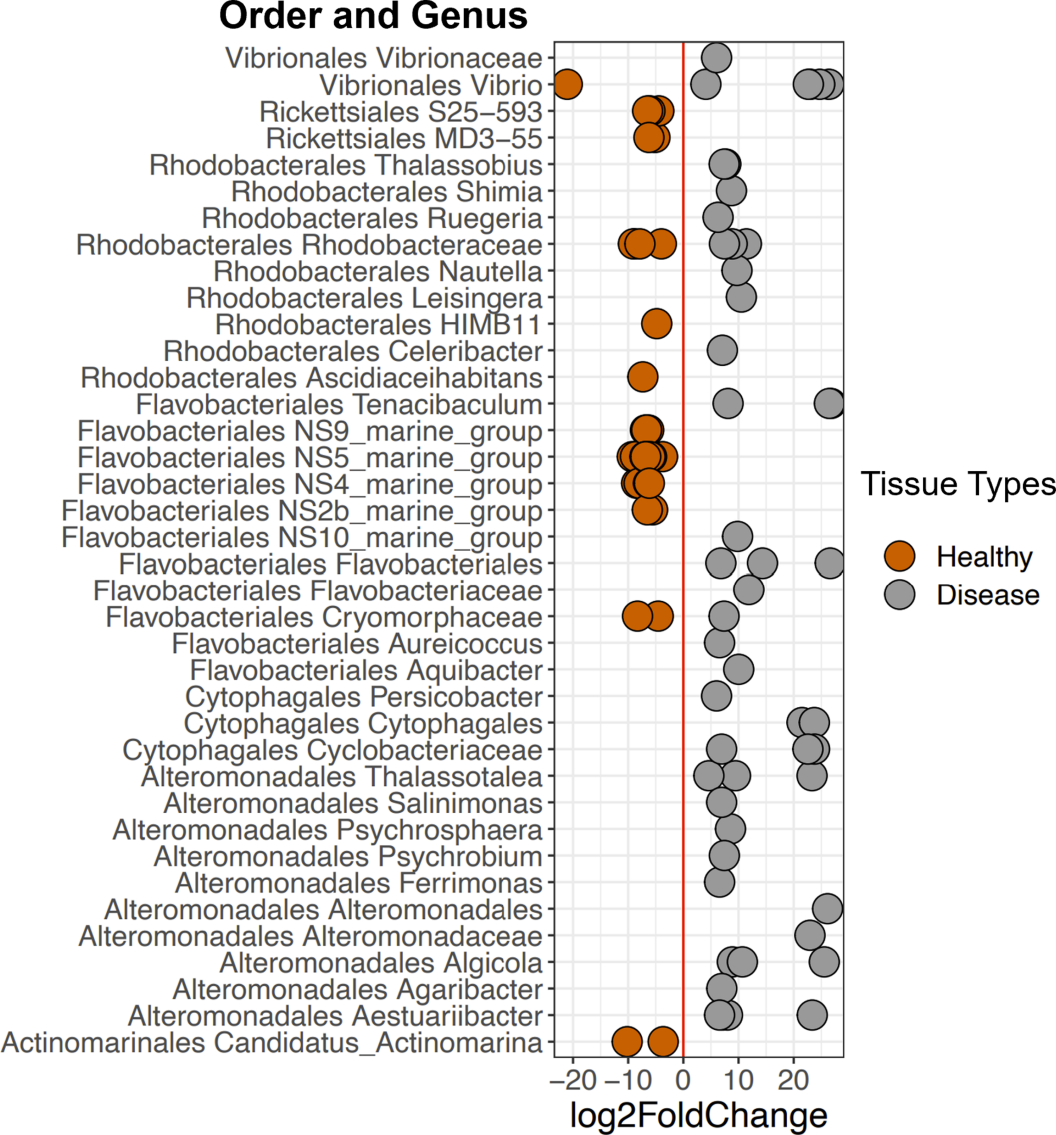
Selected ASVs that were significantly differentially abundant across Disease and Healthy tissue types for 2019 samples. Each point represents an ASV that was significantly differentially abundant in either Diseased tissue or Healthy tissues (from a healthy colony) according to the DESeq2 analysis. Point colors represent the different tissue types. There was a total of 214 ASVs that were significantly enriched (Table S2).

DESeq2 results suggest 214 ASVs that were differentially abundant among Healthy and Disease tissue types (Table S2). Several of these bacterial taxa displayed higher abundance in Disease samples compared to the Healthy 2019 tissue type (91 ASVs, Fig. 6, Table S2), including *Vibrio* (Vibrionales), *Algicola* (Alteromonadales), Flavobacteriales, *Thalassobius* (Rhodobacterales), *Leisingera* (Rhodobacterales), and an unclassified genus of Cytophagales. Others were enriched in Healthy 2019 (123 ASVs) tissues compared to Disease: MD3-55 (Rickettsiales), HIMB11 (Rhodobacterales), *Vibrio* (Vibrionales), Francisellaceae (Flancisellales), an unclassified genus of Alphaproteobacteria, *Litoricola* (Oceanospirillaes), and *Candidatus* Actinomarina (Actinomarinales). The ANCOM results reflect the same patterns and indicate that taxa significantly enriched in the Healthy colonies are also enriched in Apparently Healthy colonies compared to Disease (Fig. S3, Table S3).

Among Healthy corals, communities did not differ significantly between 2017 and 2019 (PERMANOVA: *p*-value = 0.078, R^2 ^ = 0.123, Fig. 5). This is likely due to the high relative abundance of Rickettsiales that the healthy tissue types share. When Rickettsiales was excluded from the analysis, DeSeq2 showed 13 differentially abundant ASVs (Fig. S4, Table S4). These differences may simply be due to stochastic differences in these rare taxa.

## Discussion

Diseases are leading to global declines in corals. Here we had the unique opportunity to sample the microbiome of *A. cervicornis* before and during a white band disease outbreak in an ocean-based coral nursery in Little Cayman, CI. We found Healthy and Apparently Healthy tissue microbial communities were markedly different from communities in tissues associated with Disease lesions, and Disease tissues showed a microbiome consistent with WBD and other coral diseases. Our results also suggest consistency in the community associated with Healthy tissues before and during the outbreak (at two separate time points).

We unexpectedly found few differences in Shannon diversity across tissue types, but we observed higher Inverse Simpson index values for Diseased corals compared to Healthy tissue types. This suggests that there were a similar *number* of taxa across tissue types, however *evenness* of the taxa is driving differences across the tissue types. Indeed, all healthy tissue types (2017 Healthy, 2019 Healthy and 2019 Apparently Healthy tissue on diseased colonies), showed high relative abundances of Rickettsiales, but these taxa were largely absent in diseased corals, or greatly reduced in relative abundance.

We found several bacterial groups increased in Disease tissues compared to Healthy tissues (Fig. 6), suggesting they may be part of the coral’s pathobiome. Some of these taxa have been implicated in other coral or invertebrate diseases and are likely candidates for the consortia of putative pathogens that contribute to pathogenesis, including Vibrionales, Alteromonadales, Rhodobacterales, and Cytophagales (Baker-Austin et al., 2018; Guibert et al., 2020; Welsh et al., 2017). Orders Vibrionales and Alteromonadales are groups of antagonistic Gammaproteobacteria that have previously been found in higher relative abundances on diseased corals (Rypien, Ward & Azam, 2010; Arotsker et al., 2015; Meyer et al., 2019). *Algicola* has also been observed to be in higher abundance among disease lesions in stony coral tissue loss disease (SCTLD) (Meyer et al., 2019). Additionally, a report from the Florida Department of Environmental Protection identifies *Leisingera* (Rhodobacterales), as we found in this study, as a putative pathogen in SCTLD. The strain *Leisingera sp*. McT4-56 has been isolated and utilized to test against potential probiotics for corals (Paul et al., 2021; Deutsch et al., 2022). Furthermore, inoculation of pathogenic *V. coralliilyticus* on *Montastraea cavernosa* corals also increased the mean relative abundance of Vibrionales, Rhodobacterales, and Cytophagales; suggesting ASVs in the orders Cytophagales and Rhodobacterales are opportunists (Welsh et al., 2017).

*Vibrio* is a putative pathogen that could act as a causative disease agent in Disease tissue types. The most prevalent *Vibrio* ASVs in Disease samples were the species *V. harveyi* and *V. parahaemolyticus* (based on NCBI BLAST results), both of which belong to the Harveyi clade and are commonly identified in several coral diseases (Gil-Agudelo, Smith & Weil, 2006; Luna et al., 2010; Ushijima et al., 2012), including WBD type II. *Vibrio*, as well as *Algicola*, were found consistently across tissue types and years (see Fig. S3) but were also significantly higher in abundance in Disease tissue than in either Healthy or Apparently Healthy tissues. Their presence, even in relatively low abundance, in Apparently Healthy and Healthy tissue may be a sign of future microbial instability and/or an indicator that the whole coral (areas with and without visible lesions) may be compromised (Egan & Gardiner, 2016; Kemp et al., 2018) or more susceptible to disease.

**Figure 7:**
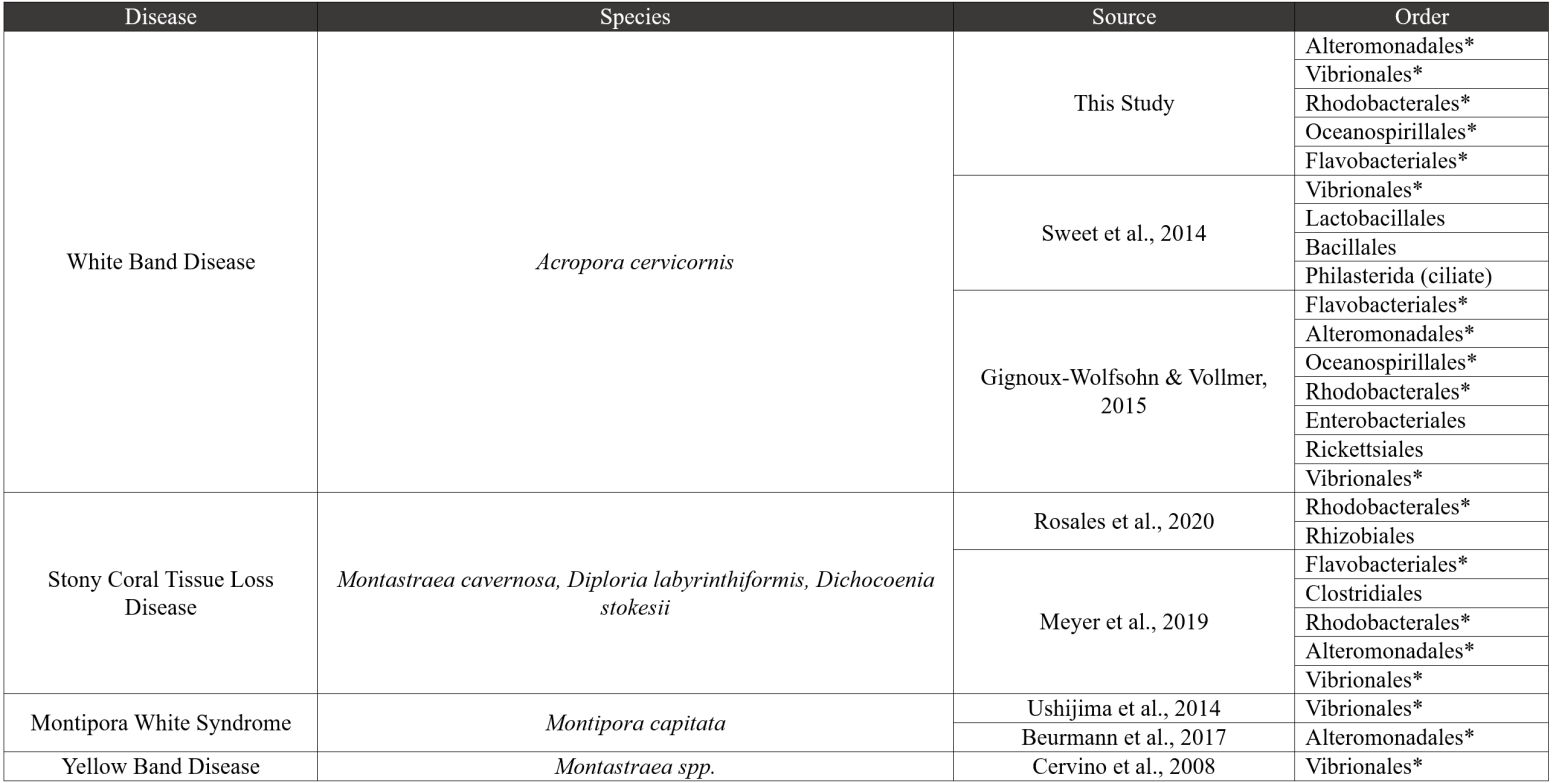
Comparative disease table. Bacterial orders from our study were compared to seven other coral disease studies. The table characterizes four diseases, and bacterial orders that are shared with our study are shown using an asterisk next to the order name.

However, it is also possible that *Vibrio* and the disease-associated taxa are a consequence of disease, rather than the cause of the disease. These disease-associated taxa may simply exist in relatively low abundance throughout the coral microbiome (Raina et al., 2009; Rypien, Ward & Azam, 2010; Peixoto et al., 2017) and then bloom during disease progression due to changes in nutrient or metabolite composition (Gignoux-Wolfsohn & Vollmer, 2015; Sweet & Bulling, 2017; Xue et al., 2017; Ochsenkühn et al., 2018; Meyer et al., 2019). Additional groups including *Tenacibaculum* (Flavobacteriales), *Thalassobius* (Rhodobacterales), *Thalassolituus* (Oceanospirillales), *Aestuariibacter* (Alteromonadales), and *Arcobacter* (Campylobacterales), were found in higher relative abundance in Disease tissues in this study. Some of these taxa are consistently associated with coral diseases, including stony coral tissue loss disease, Yellow Band Disease, and others (Fig. 7). Together, the shifts in these taxa across different diseases suggest they are general indicators of disease, and good candidates for further study to understand their roles as causal agents *versus* secondary players.

The most marked difference between tissue types is the reduction of MD3-55 in disease tissues and the abundance of MD3-55 in healthy tissues. The ASVs associated with genus MD3-55 are highly similar or identical to *Candidatus* Aquarickettsia rowheri, which has been described as an endocellular parasite (Klinges et al., 2019; Klinges et al., 2020). Here and elsewhere, Rickettsiales have been reported in samples of both healthy and diseased *Acropora* corals (and genotypes), making it unlikely that Rickettsiales are strictly pathogens (Godoy-Vitorino et al., 2017; Klinges et al., 2019; Miller et al., 2020; Palacio-Castro et al., 2022). However, there is some evidence that coral genotypes that contain higher abundances of Rickettsiales are more susceptible to WBD (Klinges et al., 2020) and can contribute to the onset or progression of WBD (Gignoux-Wolfsohn et al., 2020). We did not test this hypothesis here, but previous work has demonstrated that among healthy corals with similarly high relative abundances of this genus, ASV composition varied with coral genotype (Miller et al., 2020). This microbial genus also shows regional differences in abundance (Baker et al., 2022). Thus, based on the results here and from other studies, there are two potential (not mutually exclusive) hypotheses that warrant further study: (1) because of the high relative abundance of *Ca.* Aquarickettsia across all healthy corals, if this taxon is an indicator of disease susceptibility, our results suggest that all of the genotypes in this study are susceptible to disease; and/or (2) there are other functional roles this microbial taxon plays in healthy corals. Further study is needed to elucidate the functional role of Rickettsiales, and particularly the genus *Ca*. Aquarickettsia, in healthy *Acropora* corals.

Interestingly, we found that microbial communities in Apparently Healthy and Healthy tissue in 2019 and Healthy tissues between 2017 and 2019 were remarkably similar in the CCMI nursery. Because of the predominance of Rickettsiales in the genus *Ca.* Aquarickettsia, the microbiome of Healthy and Apparently Healthy tissues was generally stable across the two years sampled. As *Acropora* coral hosts are known to exert more control over their microbial communities than other coral genera, consistency in microbiomes is expected (Dunphy et al., 2019), until disrupted by disease. We did not see significant variation due to genotype which has been previously shown in these corals (Miller et al., 2020). This is likely because of the low sample sizes across genotypes, and/or because the variation due to disease was greater than variation attributed to genotype in healthy corals (*i.e.,* Fig. 5). As others have found, there may be interactions between genotype and healthy coral microbiomes (Fig. S2), which we did not test here and requires further study (*i.e.,* as tested in Miller et al., 2020). These results indicate that most microbial indicators of disease, for a time, are localized to the disease lesion, and in general, the healthy coral communities are stable over time. These overall patterns suggest that portions of diseased corals with visibly healthy tissue may be rescued from disease (*e.g.*, if fragmented away from disease lesions, Miller et al., 2014), although these areas of the coral may also be susceptible to disease and/or the bacteria that are putative disease agents were present in undetectable levels. In the future, following the coral (and microbial) fate of healthy colonies over time will provide a greater context to why some colonies become diseased, and some do not.

## Conclusions

Our results highlight the complexity of the coral microbiome and the pathobiome in coral health and disease as we examined the microbial diversity of *A. cervicornis* mucus across health states and time. We show a suite of microbial taxa that are consistently enriched in Disease samples, including putative pathogens like *Vibrio*. Like many other coral diseases, no single putative pathogen has been identified as a causative agent for WBD or other acute tissue loss syndromes in acroporid corals (Sweet, Croquer & Bythell, 2014; Gignoux-Wolfsohn & Vollmer, 2015). We suggest WBD may be polymicrobial, consistent with other WBD studies (Lesser et al., 2007; Sweet, Croquer & Bythell, 2014; Gignoux-Wolfsohn & Vollmer, 2015). Further research, such as infection studies, are needed to identify whether *Vibrio* and other disease-related taxa eventually contribute to the pathobiome of coral mucus and ultimately result in disease in Caymanian *Acropora cervicornis*.

Healthy tissue microbial communities were consistent before and after the onset of disease and were not different from Apparently Healthy tissues. This pattern was driven by the high relative abundance of Rickettsiales genus *Ca.* Aquarickettsia in all Healthy tissues, aligning with previous studies (Klinges et al., 2020; Aguirre et al., 2022; Williams et al., 2022). This taxon is associated with healthy coral here and further studies should be conducted to further elucidate the role of Rickettsiales in the *A. cervicornis* microbiome and coral health. Because we observed no clear differences in the taxa associated with Apparently Healthy tissues and Healthy colonies, a potential disease mitigation method in nurseries may be trimming away the diseased tissue, however this will require further study before wide scale implementation.

Massive population declines among *Acropora* corals throughout the Caribbean have driven the establishment of coral nurseries and restoration programs (Lirman & Schopmeyer, 2016; Schopmeyer et al., 2017; Bayraktarov et al., 2020). Due to their fast growth and importance to marine ecosystems, *Acropora* corals have been the focus of many restoration efforts in the Caribbean with numerous studies beginning to emerge from nurseries and restoration sites (Miller et al., 2019). Coral nurseries are unique in that individual corals, often with known genotypes, can be tracked and observed throughout life stages and over time. Following microbial markers among corals is especially important to understand coral responses to environmental perturbations such as disease. Few tools have been developed to effectively manage coral disease. If microbes significantly contribute to disease resistance, these corals could be used to leverage restoration efforts and potentially improve outplanting and survival success (Schopmeyer et al., 2012; Rosales et al., 2019). Our results add to the larger body of literature describing coral diseases as polymicrobial, and we are the first to describe the microbiome associated with white band disease in the Cayman Islands. Our work extends the foundational knowledge of healthy and disease microbial communities that can be used to understand disease dynamics in *Acropora* corals and to develop future mitigation strategies for disease resistance and resilience in coral conservation.

##  Supplemental Information

10.7717/peerj.15170/supp-1 Figure S1 Rarefaction curves Each line represents a different sample. Our data shows each line (sample) shows saturation, indicating we have captured the richness in this dataset. The rarefaction curves were generated in vegan (Oksanen et al., 2020) using a step (e.g., number of reads added per step) equal to 200.

10.7717/peerj.15170/supp-2 Table S1 Quality filtering of ASVs performed with DADA2 and phyloseq Sample names and their associated metadata including quality filtered reads and number of sequences per sample.

10.7717/peerj.15170/supp-3 Figure S2 PCoA of each sample from each treatment and year by group PCoA on the bray Curtis dissimilarities from relative abundances of each ASV in each sample. Each point represents a coral tissue sample, shapes represent tissue type (coral health) and year and facet indicate group. Group is defined by samples from the same genotype on the same frame and the group is written at the top of each graph. Ellipses and color are based on genotype.

10.7717/peerj.15170/supp-4 Figure S3 Relative abundance of genera significantly different based on the ANCOM results. Data shows consistency with the DeSeq analysis Mean relative abundance ±SE of specific ASVs that significantly differed based on tissue type during the disease outbreak according to the ANCOM analysis (please note differences in Y-axis scales in smaller plots). Points represent individual ASVs, and each small plot is grouped by genus and family. Point colors represent the different tissue types and are ordered as represented in the legend in each small plot. *Vibrio, Thalassotalea, Thalassolituus, Catenococcus*, Flavobacteriales (lowest identifiable taxonomic level) and *Algicola* have a higher relative abundance in disease samples. *Candidatus* Aquarickettsia rohweri (MD3-55) and some common seawater microbes show higher relative abundance in both healthy and apparently healthy samples. Full ANCOM results are in Table S3.

10.7717/peerj.15170/supp-5 Figure S4 ASVs that were significantly differentially abundant across Healthy 2017 and Healthy 2019 tissue types without Rickettsiales Each point represents an ASV that was significantly differentially abundant in either Healthy 2017 tissue or Healthy 2019 tissues according to the DESeq2 analysis. The Order Rickettsiales was excluded from the analysis to view less abundant groups. Point colors represent the different tissue types. There was a total of 13 ASVs that were significantly enriched (Table S5).

10.7717/peerj.15170/supp-6 Table S2 DeSeq2 Analysis of Healthy 2019 VS Disease 2019

10.7717/peerj.15170/supp-7 Table S3 ANCOM results for a 90% cutoff of 2019 samples comparing Apparently Healthy, Healthy and Disease

10.7717/peerj.15170/supp-8 Table S4 DeSeq2 analysis Healthy 2017 vs Healthy 2019 with no Rickettsiales

## Additional Information and Declarations

### Field Study Permissions

The following information was supplied relating to field study approvals (*i.e.*, approving body and any reference numbers): This work was completed under permits approved by the Cayman Islands Department of Environment for Anya Brown (mucus sampling) in 2019, and for Carrie Manfrino and Thomas Frazer in 2018-2020 (the ongoing coral nursery permit), and a CITES export permit approved in May 2019.

### Data Availability

The following information was supplied regarding data availability: The code is available at GitHub and Zenodo:
https://github.com/anyabrown/SchulDiseaseAcervicornis. Anya Brown. (2023). anyabrown/SchulDiseaseAcervicornis: Schul PeerJ 2023 (Version Schul2023). Zenodo. https://doi.org/10.5281/zenodo.7802849. The 2019 samples are available at NCBI BioProject: PRJNA679809.
