# Supplementary figures and images for: Concordance of microbial and visual health indicators of white-band disease in nursery reared Caribbean coral Acropora cervicornis
Source: PeerJ. 2023 Jun 21;11:e15170. doi: 10.7717/peerj.15170 (PMC10290447; doi:10.7717/peerj.15170)

# Rarefaction Curve for all samples

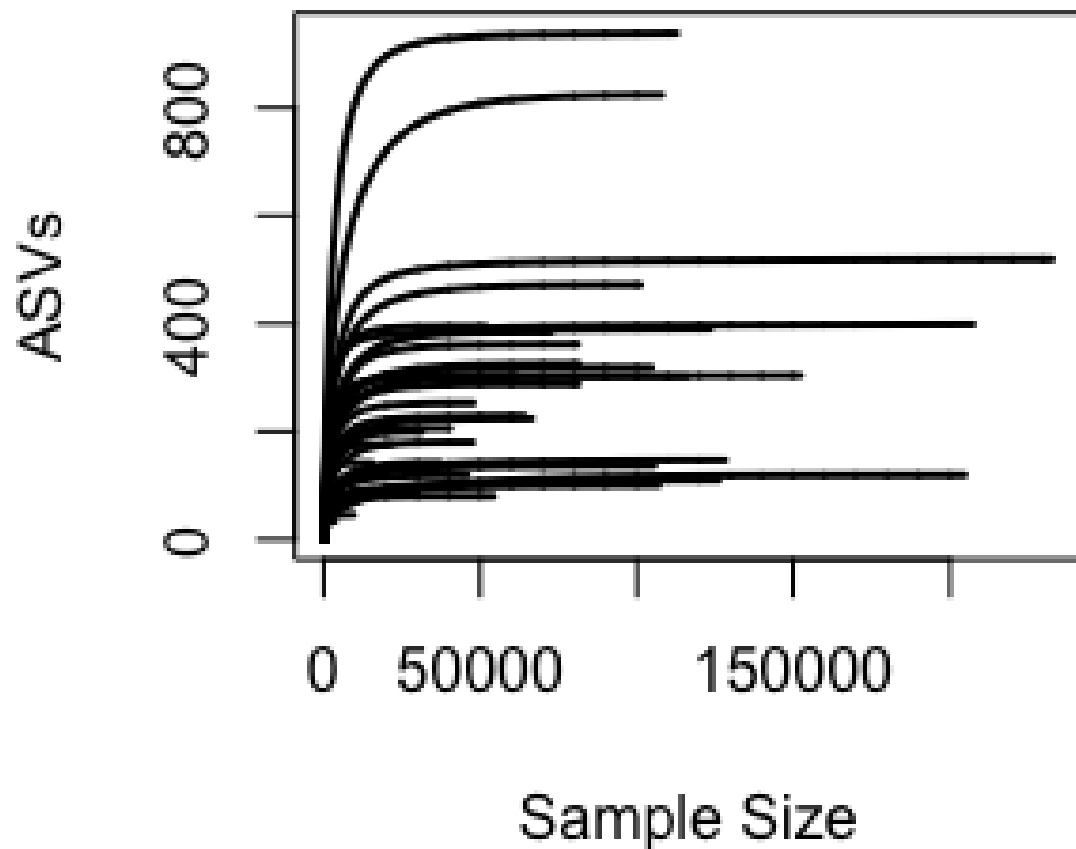

Supplement: Figure S1 — Each line represents a different sample. Our data shows each line (sample) shows saturation, indicating we have captured the richness in this dataset. The rarefaction curves were generated in vegan (Oksanen et al., 2020) using a step (e.g., number of reads added per step) equal to 200. [file peerj-11-15170-s001.pdf]

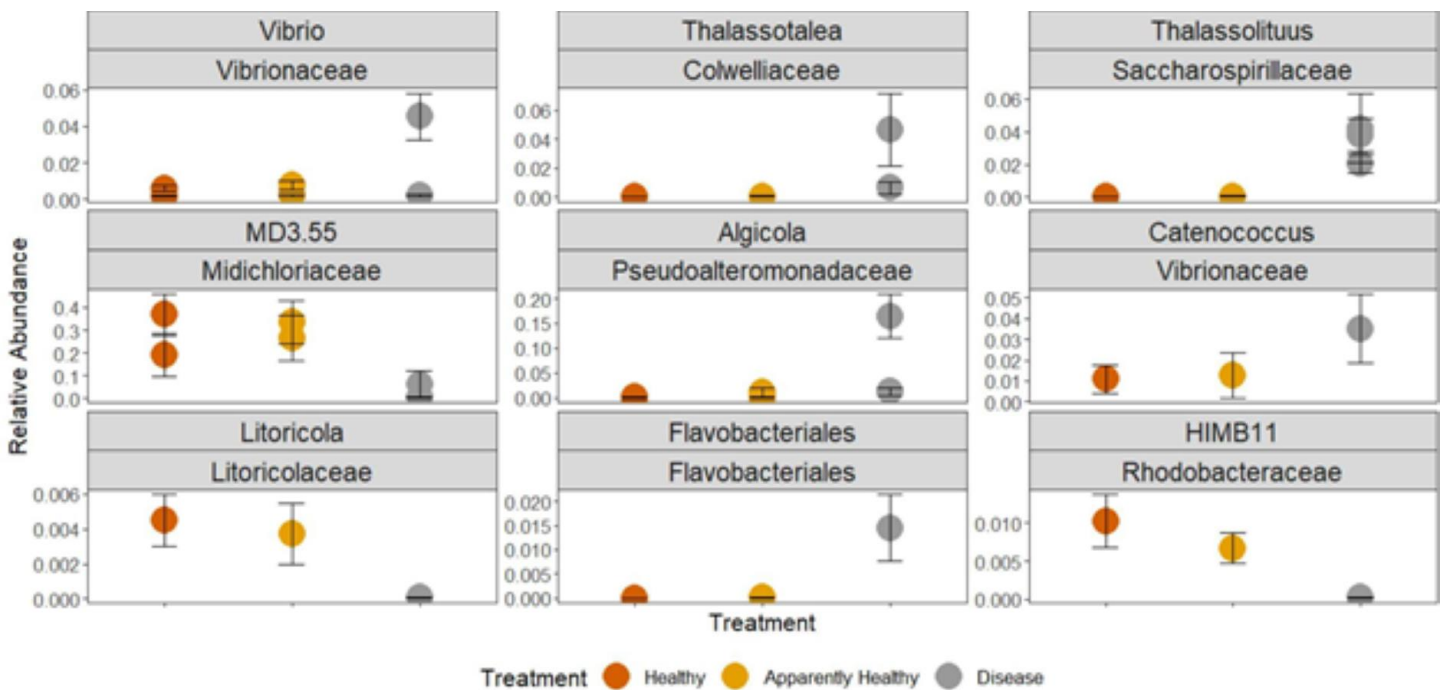

Supplement: Figure S3 — Mean relative abundance ±SE of specific ASVs that significantly differed based on tissue type during the disease outbreak according to the ANCOM analysis (please note differences in Y-axis scales in smaller plots). Points represent individual ASVs, and each small plot is grouped by genus and family. Point colors represent the different tissue types and are ordered as represented in the legend in each small plot. Vibrio, Thalassotalea, Thalassolituus, Catenococcus, Flavobacteriales (lowest identifiable taxonomic level) and Algicola have a higher relative abundance in disease samples. Candidatus Aquarickettsia rohweri (MD3-55) and some common seawater microbes show higher relative abundance in both healthy and apparently healthy samples. Full ANCOM results are in Table S3. [file peerj-11-15170-s004.pdf]

# Order and Genus

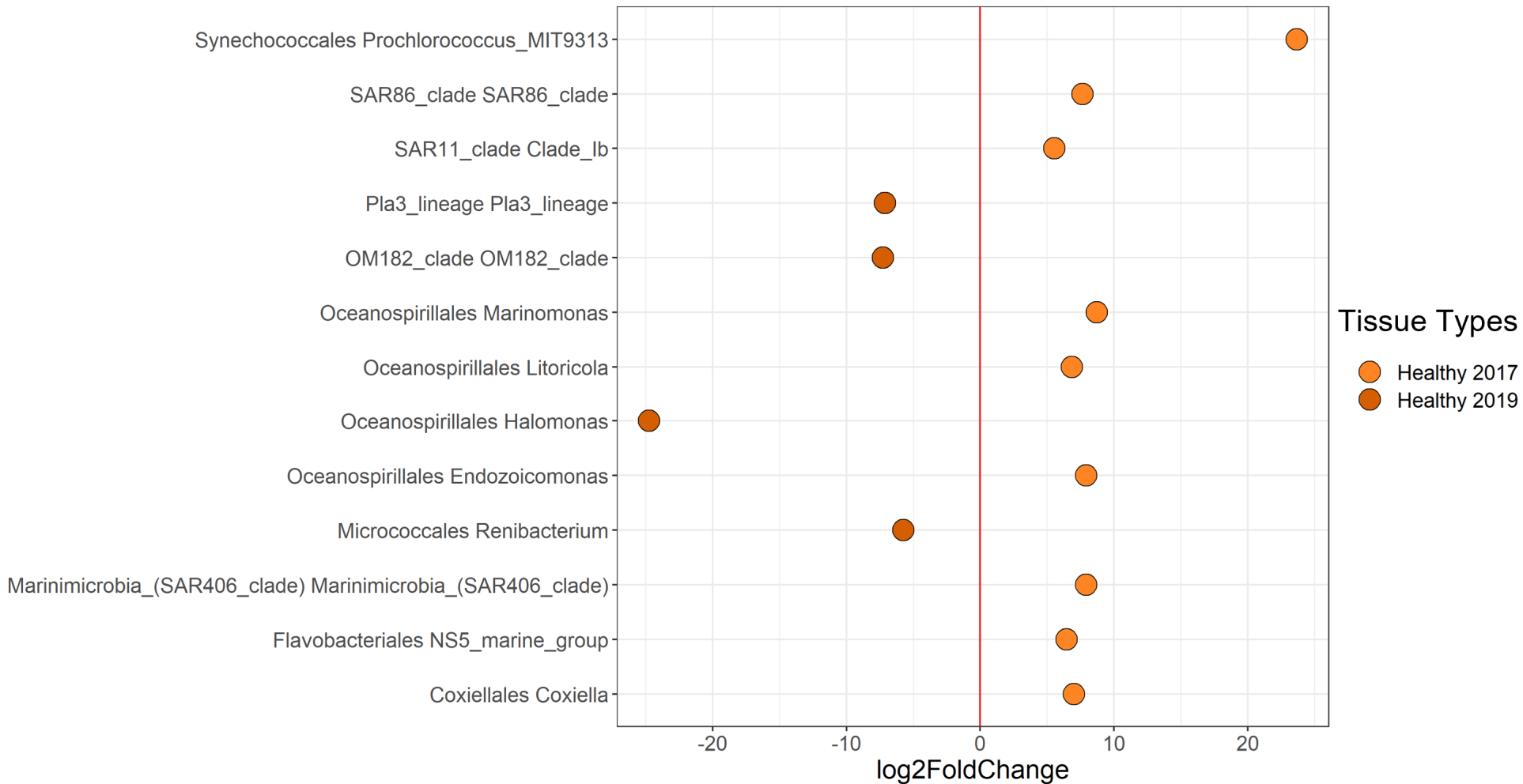

Supplement: Figure S4 — Each point represents an ASV that was significantly differentially abundant in either Healthy 2017 tissue or Healthy 2019 tissues according to the DESeq2 analysis. The Order Rickettsiales was excluded from the analysis to view less abundant groups. Point colors represent the different tissue types. There was a total of 13 ASVs that were significantly enriched (Table S5). [file peerj-11-15170-s005.pdf]
