# Supplementary material for: Concordance of microbial and visual health indicators of white-band disease in nursery reared Caribbean coral Acropora cervicornis
Source: PeerJ. 2023 Jun 21;11:e15170. doi: 10.7717/peerj.15170 (PMC10290447; doi:10.7717/peerj.15170)
Supplement: Figure S2 — PCoA on the bray Curtis dissimilarities from relative abundances of each ASV in each sample. Each point represents a coral tissue sample, shapes represent tissue type (coral health) and year and facet indicate group. Group is defined by samples from the same genotype on the same frame and the group is written at the top of each graph. Ellipses and color are based on genotype. [file peerj-11-15170-s003.pdf]

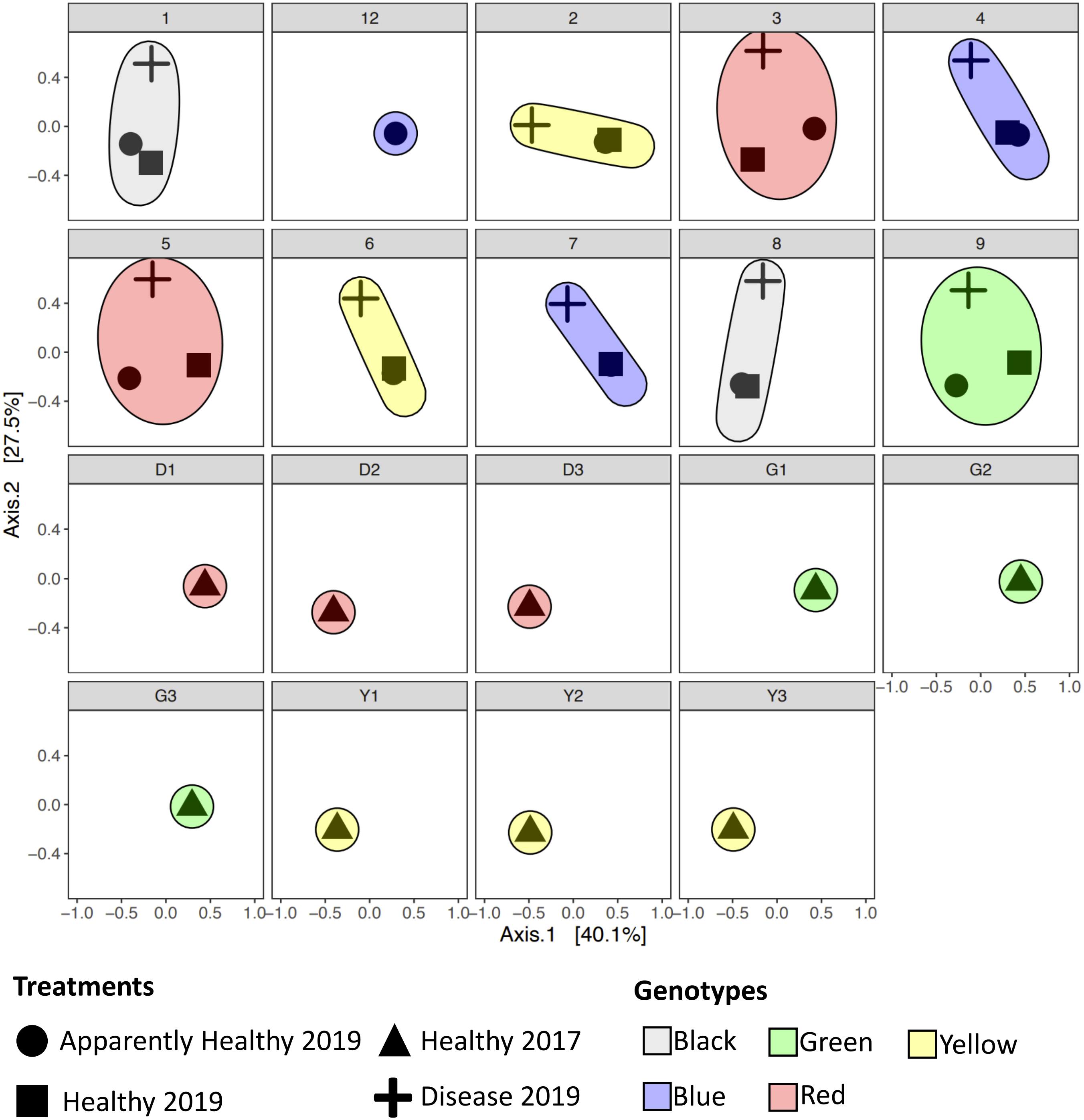
1
12
2
3
4
5
6
7
8
9
D1
D2
D3
G1
G2
G3
Y1
Y2
Y3
Axis.2 [27.5%]
Axis.1 [40.1%]
Treatments
Apparently Healthy 2019
Healthy 2017
Healthy 2019
Disease 2019
Genotypes
Black
Green
Yellow
Blue
Red
